# Evaluation on Early Strength Development of Concrete Mixed with Non-Sintered Hwangto Using Ultrasonic Pulse Velocity

**DOI:** 10.3390/ma16216850

**Published:** 2023-10-25

**Authors:** Youngjin Nam, Keesin Jeong, Wonchang Kim, Hyeonggil Choi, Taegyu Lee

**Affiliations:** 1 Department of Fire and Disaster Prevention, Semyung University, Jecheon 27136, Republic of Korea; aaa9480@naver.com (Y.N.); jks@semyung.ac.kr (K.J.); kimwc69082@gmail.com (W.K.); 2 School of Architecture and Civil Engineering, Kyungpook National University, Daegu 41566, Republic of Korea

**Keywords:** non-sintered hwangto, ultrasonic pulse velocity, compressive strength, prediction model of compressive strength, correlation

## Abstract

Currently, in order to reduce the greenhouse gases of global warming, research on alternative cement materials is being actively conducted in the construction industry to reduce cement use, and it is judged to be important to evaluate the timing of form removal for the initial age. Therefore, in this study, we evaluated the initial mechanical properties of concrete in which cement was partially replaced with non-sintered hwangto (NHT). Specimens without NHT (namely, normal mortar (NM) and normal concrete (NC)) and specimens with NHT (namely, non-sintered hwangto mortar (HTM) and non-sintered hwangto concrete (HTC)) were prepared. NHT was substituted for 15% and 30% of cement. Two water-to-binder (W/B) ratios, 41% and 33%, were used to analyze the variation in the mechanical properties according to the cement and NHT content per unit volume of concrete. The compressive strength and ultrasonic pulse velocity (UPV) were measured. Experimental results indicated that compressive strength decreased with an increase in NHT content. The mortar with NHT substitution rates of 15% and 30% exhibited higher UPV than NM at a W/B ratio of 41%, in contrast to the behavior observed for concrete. The UPVs of most specimens were similar regardless of the NHT substitution rate. The correlation between the compressive strength and UPV of HTC was analyzed, and therefrom, exponential equations with a high correlation coefficient (R^2^) were proposed for strength prediction; the resulting predictions were compared with the results of previous compressive strength prediction models.

## 1. Introduction

The construction industry is responsible for 41% of total CO_2_ emissions. In particular, CO_2_ emissions from the cement firing process account for approximately 8.8% [1]. Methods to reduce greenhouse gas emissions have been widely investigated since the Kyoto Protocol [2]. In the construction industry, cement substitutes, such as fly ash, blast furnace slag, and red mud, have been investigated to reduce CO_2_ emissions due to the cement firing process [3,4,5,6,7,8]. According to data provided in EN 197-1:2011 [9], approximately 0.49 tons of carbon dioxide is generated when producing 1 ton of Portland cement. It can be seen that the amount of carbon dioxide reduced varies depending on the replacement ratio of 0.34 to 0.48 tons when producing 1 ton of cement using fly ash and blast furnace slag, which are existing cement mixing materials. In addition, in this experiment, the study was conducted because when replacing hwangto as a cement binder, the same carbon dioxide reduction as that of the existing binder can be expected. Research to reduce carbon dioxide is also underway in Europe, and recent research on using clay as a cement replacement material has been continuously conducted in Europe. Clay is a cement replacement material for hwangto and is expected to have similar mechanical properties. It is believed that through research on clay and hwangto, the construction industry will be able to reduce costs by reducing cement, and it will be an opportunity to change people’s perception of it as an eco-friendly material. In addition, this study is expected to be helpful in research on early age related to soils such as clay and hwangto.

In particular, hwangto has been studied since the 2000s. Hwangto is easy to obtain because it is found in approximately 10% of the ground surface worldwide and has a chemical composition similar to existing cement admixtures, such as fly ash and blast furnace slag [10,11,12]; moreover, hwangto offers numerous advantages, such as temperature/humidity control and deodorizing functions [13]. Hence, hwangto has attracted increasing attention as an eco-friendly building material. However, the use of hwangto has been limited to low-rise residential buildings owing to low strength development and durability degradation. Compared to concrete using only cement, as the ratio of hwangto in concrete mixed with hwangto increases, cracks increase and workability, air volume, and setting speed decrease [14,15,16,17,18]. The low strength development of concrete mixed with hwangto is attributed to the approximately 6% decrease in volume due to the cohesion between particles when hwangto reacts with water, which leads to cracks [19].

**Figure 1:**
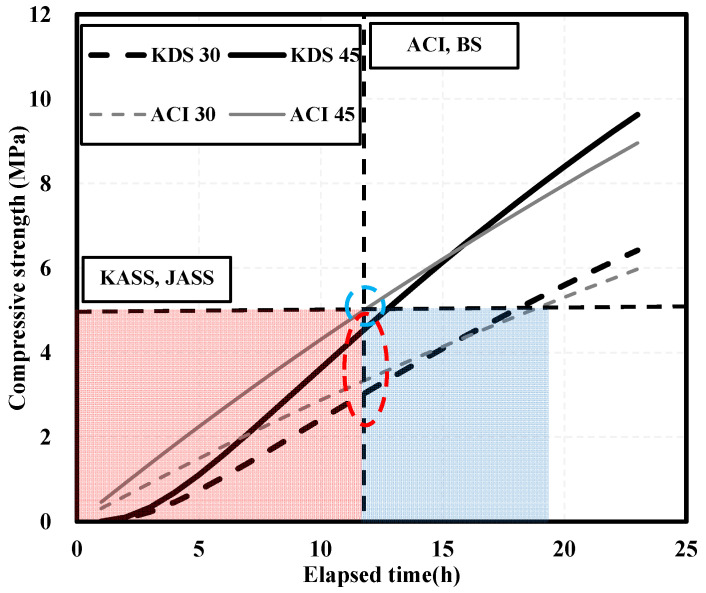
Regulation of mold removal points.

Low strength development may have a considerable impact on the construction period and quality in the early construction stage. In the early construction stage, it is important to evaluate the timing of form removal, and if a form collapse accident occurs during the construction process, it can result in significant human and property damage. In particular, the low strength of hwangto can have a significant impact on the construction period and quality in the early construction stages. Because concrete mixed with hwangto has lower strength compared to concrete mixed with 100% cement, evaluation at the time of form removal, such as with existing concrete, may cause problems such as form collapse. The vertical formwork demolding point is specified as 5 MPa in KASS and JASS and as 12 h after concrete pouring in the ACI and BS standards [20,21,22,23]. In each specification, the evaluation for demolding concrete vertical formwork is divided into compressive strength and curing time. Figure 1 shows the estimations of strength development over time for concrete with target strengths of 30 and 45 MPa [22,24].

The red shaded area is the part that did not reach the strength of 5 MPa and the time of 12 h, which is the point at which the vertical form was demolded, and the blue shaded area is the part that only reached 12 h. In addition, the blue dashed circle is the part where the form demolding strength and time have been reached, and the red dashed circle is the part where the form demolding strength has not been reached. After 12 h, which is the formwork demolding point, only the ACI estimation equation reached 5 MPa for concrete with a target strength of 45 MPa, whereas the other estimation equation did not. Since the difference in strength over time is significant in evaluating the formwork demolding point of concrete, confirming a developed strength of 5 MPa is considered a safe and quantitative decision criterion. Therefore, the formwork demolding point was evaluated using strength in this study [25].

The compressive strength of concrete is part of the quality index and is also an index that predicts various strengths of concrete. It is believed that overlooking the compressive strength of concrete can have a direct impact on cost, construction period, and quality. It is necessary to evaluate the compressive strength at an early age in an accurate and quantitative manner. Fracture tests on specimens are necessary for an accurate evaluation of strength development in concrete. However, identifying the accurate strength development point is challenging because the mechanical properties of concrete can be affected by environmental factors, such as temperature and weather, and monitoring the concrete strength development mechanism is difficult. To address this problem, studies have investigated the identification of strength development point using ultrasonic pulse velocity (UPV), which is a non-destructive method [4,26,27,28]. UPV is an easy and rapid technique to obtain results with high accuracy and, thus, facilitates the monitoring of density change and cracking in specimens during concrete setting and curing. An accurate evaluation of the strength development is necessary when new materials, such as non-sintered hwangto (NHT), are used in concrete.

In existing studies, the correlation between the compressive strength of concrete and UPV was expressed as an exponential function or a linear function. Looking at the correlation between compressive strength and UPV, the degree of increase in compressive strength and UPV is minimal at first, but as time passes, compressive strength increases rapidly compared to UPV. When the correlation between the compressive strength of concrete and UPV was expressed as a linear function and as an exponential function, the correlation coefficient of the exponential function was high. Additionally, because the correlation graph between the compressive strength of concrete and UPV is similar to a quadratic equation, it is expressed as an exponential function. Therefore, in this study, hwangto, an eco-friendly material, was used as a construction material and compressive strength and UPV were measured from the initial age over time according to the replacement rate of hwangto as a cement substitute material. We would like to propose a strength prediction equation in the form of an exponential function by analyzing the compressive strength of concrete mixed with hwangto and the UPV results through correlation.

## 2. Materials and Methods

### 2.1. Outline of the Experimental Procedure

Table 1 shows the experimental parameters used in this study. Specimens without NHT—namely, normal mortar (NM) and normal concrete (NC)—and specimens containing NHT—namely, non-sintered hwangto mortar (HTM) and non-sintered hwangto concrete (HTC)—were used. The specimens were cylindrical. The water-to-binder (W/B) ratio was set to 33% and 41%. The specimens were cured in a constant temperature (20 ± 2 °C) and humidity (60 ± 5%) chamber. The compressive strength and UPV were measured during the 24 h curing period in accordance with ASTM C39/C39M [29] and ASTM C597 [30] to determine the strength at the formwork demolding point during the initial stages.

Table 2 shows the experimental plan. The measurement items are compressive strength and UPV. The compressive strength of concrete was measured at 120 min intervals for 6 to 24 h, while the UPV of concrete and mortar was measured at 30 min intervals for 6 to 12 h and at 60 min intervals for 12 to 24 h. Three samples of each specimen were used, and the average compressive strength and UPV values were used in the analyses. The surface of the concrete was smoothed to measure the compressive strength and UPV values consistently. In the case of compressive strength, the average of the values of three test specimens is shown in the graph, and in the case of UPV, the average of the values of two test specimens is shown in the graph. To accurately measure UPV, vacuum grease was applied to the side surface of the concrete to ensure close contact between the probe and the test object. This UPV measures the time for a signal to arrive from the transmitting converter to the receiving converter. When the signal reaches the receiving converter, the speed appears on the display and the experiment can be measured through that value [31].

### 2.2. Materials

Table 3 shows the physical properties of the materials used in this study. Type I ordinary Portland cement (OPC) with a density of 3.15 g/cm^3^ and a fineness of 3200 cm^2^/g was used as the cement. Previous studies have shown that sintered hwangto is not suitable for reducing greenhouse gas emissions due to CO_2_ generation during sintering. Hence, herein, NHT with a density of 2.5 g/cm^3^ and a fineness of 3300 cm^2^/g was used [32]. For the coarse aggregate, crushed granite aggregate with a density of 2.68 g/cm^3^, fineness modulus of 7.03, absorption of 0.68%, and maximum size of 20 mm was mixed. For the fine aggregate, river sand with a density of 2.54 g/cm^3^, fineness modulus of 2.54, and absorption of 1.6% was mixed. Polycarboxylic-based acid, a superplasticizer, was mixed to ensure workability.

Table 4 shows the chemical properties of OPC and NHT. For cement, the content of CaO was higher than the SiO_2_ and Al_2_O_3_ content. In contrast, the SiO_2_ and Al_2_O_3_ content in NHT was higher than that of CaO. The chemical composition of NHT is similar to that of conventional cement admixtures [10,11,12].

### 2.3. Mixing Proportions

Table 5 shows the mixing proportions of NC and HTC used in this study. NHT was substituted for 15% and 30% of cement, and two different W/B ratios, 0.41 and 0.33, were used. In the column ‘MIX ID’, the number after NC and HTC indicates the W/B ratio, and ‘-15’ and ‘-30’ represent the NHT proportion substituted for cement. As for coarse aggregate (G) and fine aggregate (S), similar amounts were mixed at the same W/B ratio.

Recently, the target strengths of 45 MPa and 30 MPa have been continuously used in the construction industry, and W/B 33% and W/B 41% were set to represent the target strengths of 45 MPa and 30 MPa. Existing research results suggest that using ash and blast furnace slag as cement binders does not have a significant impact on the target strength of concrete. Therefore, in this study, experiments were conducted with NHT replacement content set at 15% and 30%.

## 3. Results and Discussion

### 3.1. Early-Age Compressive Strength of NC and HTC

**Figure 2:**
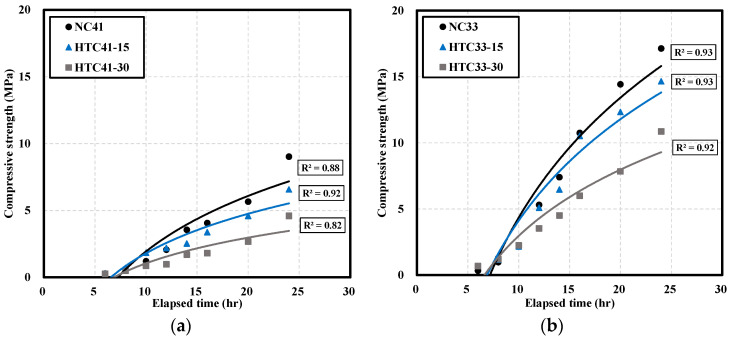
Compressive strength of NC and HTC: (**a**) W/B 41%; (**b**) W/B 33%.

Figure 2 shows the early-age compressive strength of NC and HTC. Initially, the compressive strength increased in the form of a high slope, but the rate of increase in the compressive strength decreased over time. The R^2^ values of the exponential and linear functions were significantly lower than those of the logarithmic function, and the trends of the graph of compressive strength over time were consistent with the logarithmic function, so the trend line was expressed as a logarithmic function. At a W/B ratio of 41% (Figure 2a), the strength of HTC41-15 was 37% higher than that of NC after 8 h. The strengths of NC41 and HTC41-15 after 24 h surpassed the minimum strength of 5 MPa for formwork demolding. HTC41-30 developed a strength of 4.6 MPa, which was 49% lower than that of NC41.

At a W/B ratio of 33% (Figure 2b), the strength development until 10 h was similar. After 12 h, the strength of HTC33-30 was 34% lower than that of NC33, and the difference increased over time. HTC33-15 developed a similar strength to NC33 until 16 h; however, after 20 h, its strength was 15% lower than that of NC33. Compressive strength decreased as the NHT content increased. The difference in compressive strength decreased with a decrease in the W/B ratio owing to an increase in cement content.

Within 24 h, among the test specimens with W/B 33% and W/B 41%, the rest except HTC41-30 showed 5 MPa, which is the demolding strength of the vertical formwork. This trend can be confirmed that, as shown previously, the expression of compressive strength decreases as the substitution rate of NHT increases. This result is believed to be because NHT plays a greater role in filling the voids between hydration products during the cement hydration reaction at an early age [33]. Additionally, as shown in Table 4, the chemical composition of cement and NHT are different, and the CaO content is significantly lower than that of cement. Since the CaO content directly affects the increase in compressive strength, it is believed that there is a risk of a collapse accident if the mold demolding time for NC and HTC is the same [34].

### 3.2. Early-Age Mortar and Concrete Ultrasonic Pulse Velocity

**Figure 3:**
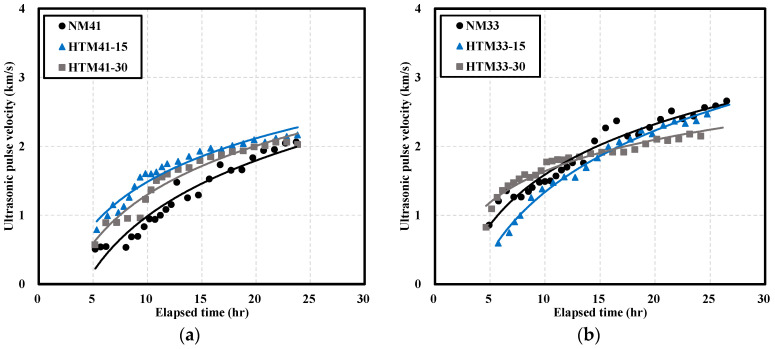
Ultrasonic pulse velocity of NM and HTM: (**a**) W/B 41%; (**b**) W/B 33%.

Figure 3 shows the UPV at different W/B ratios. Figure 3a shows the UPV of mortar when the W/B ratio was 41%. The initial difference in UPV was significant. After 6 h, the UPV values of HTM41-15 and HTM41-30 were, respectively, 0.63 and 0.39 km/s higher than that of NM41. The difference between the UPVs of NM41 and HTM41 decreased over time. The UPV of HTM was higher than that of NM. Table 6 shows the SEM images of NM and HTM at early ages. Sem image was taken of the test object at 1.5k magnification. After 6 h, HTM-15 had the lowest porosity. However, over time, NM had the highest density. As the NHT content increases, the degree of decrease of the number of pores decreases over time, and it is believed that the number of pores has a great influence on the development of the compressive strength of concrete. The higher UPV of HTM than that of NM (Figure 3a) can be attributed to the higher density of HTM at early ages [35,36].

When the W/B ratio was 33% (Figure 3b), the UPV of HTM33-30 (the HTM sample with the highest NHT substitution) was similar to that of NM33 at early ages. The UPV of HTM33-15 was 0.61 km/s, which was 30% lower than that of NM33. The UPVs of NM33 and HTM33-15 gradually turned similar while the UPV of HTM33-30 was lower. At 24 h, the UPV of HTM33-30 was 2.22 km/s, 12% lower than that of NM, and the slope of the trend line of HTM33-30 was lower than that of NM.

**Figure 4:**
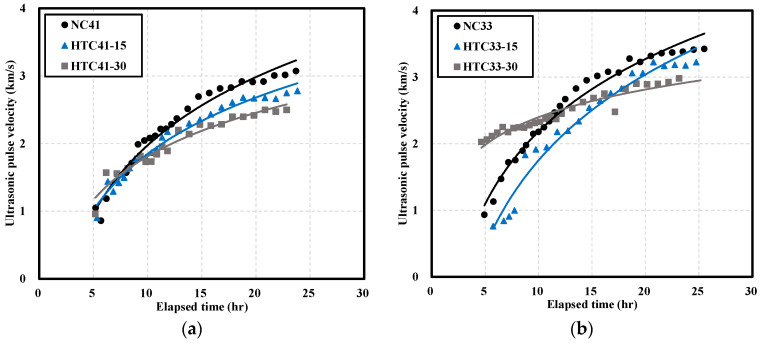
Ultrasonic pulse velocity of NC and HTC: (**a**) W/B 41%; (**b**) W/B 33%.

Figure 4 shows the UPV of NC and HTC at different W/B ratios. When the W/B ratio was 41% (Figure 4a), the UPVs of NC and HTC were initially similar. After 24 h, the UPVs of HTC41-15 and HTC41-30 were 2.78 and 2.50 km/s, respectively; these values were, respectively, 10% and 19% lower than that of NC41.

When the W/B ratio was 33% (Figure 4b), the initial UPV of HTC33-30 was more than twice higher than those of NC33 and HTC33-15. With increasing time, the UPVs of NC33 and HTC33-15 reached similar values. However, the increase in UPV of HTC33-30 over time was lower, and the UPV of HTC33-30 was 14% lower than that of NC33. The change in UPV with time was similar to that of the mortar with a W/B ratio of 33%.

**Figure 5:**
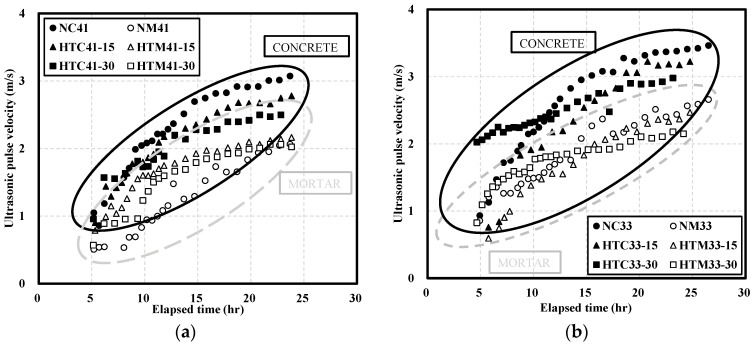
Comparison of ultrasonic pulse velocity of mortar and concrete: (**a**) W/B 41%; (**b**) 33% W/B.

Figure 5 shows the UPV of mortar and concrete over time. When the W/B ratio was 41% (Figure 5a), the UPV of mortar ranged from 0.51 to 2.17 km/s whereas that of concrete ranged from 0.86 to 3.07 km/s. The UPV of mortar was 42% lower than that of concrete.

When the W/B ratio was 33% (Figure 5b), the UPV of mortar ranged from 0.60 to 2.66 km/s whereas that of concrete ranged from 0.76 to 3.46 km/s. The UPV of mortar was 32% lower than that of concrete. Over time, the UPV of both mortar and concrete increased. At W/B ratios of 41% and 33%, the UPV of mortar was lower than that of concrete. It is believed that the reason why the UPV of mortar is lower than that of concrete is because there is no coarse aggregate in mortar [37,38].

**Figure 6:**
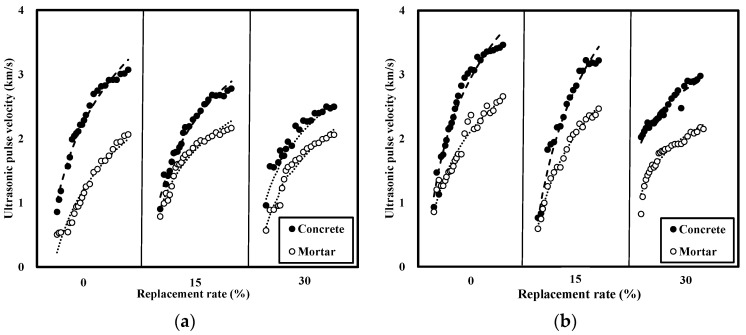
Comparison of ultrasonic pulse velocity of mortar and concrete: (**a**) W/B 41%; (**b**) W/B 33%.

Figure 6 shows the difference between the UPVs of mortar and concrete for different NHT substitution proportions. In contrast to other specimens, at a W/B ratio of 33% (Figure 6b), HTC33-30 exhibited a large initial difference of 1.21 km/s. Among the concrete specimens, NC33 showed the highest UPV of 3.46 km/s and HTC41-30 showed the lowest velocity of 2.50 km/s. The UPV decreased with an increase in the NHT proportion and the W/B ratio [39].

It can be seen from the graph that UPV shows a higher value in HTC compared to NC in the early stages of life. This trend is similar to the previous compressive strength, and it is believed that the UPV was high for a certain period of time as it played a role in filling some of the NHT voids in the voids created between cement hydration products [33]. Additionally, as the age increases, the amount of internal voids decreases as the amount of cement hydration products increases, and as the replacement ratio of NHT increases, the lower UPV appears. Looking at these results, it can be expected that the reactivity of NHT is weak at the early age.

### 3.3. Correlation between the Compressive Strength and Ultrasonic Pulse Velocity of NC and HTC

**Figure 7:**
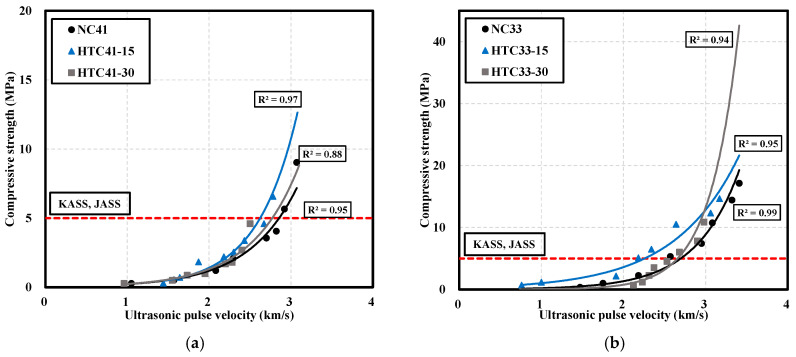
Correlation between compressive strength and ultrasonic pulse velocity: (**a**) W/B 41%; (**b**) W/B 33%.

Figure 7 shows the correlation between the compressive strength and UPV of NC and HTC according to age. In this study, the compressive strength and ultrasonic speed of NC, HTC-15, and HTC-30 at W/B 33% and W/B 41% were listed by age and the correlation was analyzed. In the case of compressive strength, there was a continuous increase over time, but in the case of ultrasonic speed, it was confirmed that the increase rate decreased over time. Previous researchers showed the correlation between compressive strength and UPV through a linear function, but a low correlation coefficient was found. However, the exponential function showed a high correlation coefficient. In addition, when the correlation between the compressive strength of concrete and UPV was graphed, it showed a tendency to be proportional to the form of a quadratic equation, so the compressive strength prediction equation was expressed using an exponential function [40]. The minimum compressive strength for formwork demolding at a W/B ratio of 41% is 5 MPa (Figure 7a). The UPV of NC41 at the demolding point was 2.92 km/s after 20 h, and that of HTC41-15 was 2.78 km/s after 24 h. However, the strength of HTC41-30 did not reach 5 MPa within 24 h. The correlation coefficients for NC41, HTC41-15, and HTC41-30 were 0.95, 0.97, and 0.88, respectively.

When the W/B ratio was 33% (Figure 7b), the compressive strengths of NC33 and HTC33-15 surpassed 5 MPa in 12 h, and their respective UPVs were 2.57 and 2.18 km/s. HTC33-30 surpassed 5 MPa after 16 h and its UPV was 2.68 km/s. At a W/B ratio of 33%, the correlation coefficient was close to 0.95 or higher and the correlation coefficient was higher than that at a W/B ratio of 41%.

In this study, the prediction equations for compressive strength development at an early age through the correlation between the compressive strength of concrete and UPV are (1), (2), and (3), and are expressed in the form of an exponential function. To compare with existing researchers’ prediction equations for the correlation between compressive strength and UPV, Table 7 [21,41,42,43] summarizes the existing prediction equations.

(1) $$Fc=0.0332\times exp\left(1.8182\times Vp\right)$$

(2) $$Fc=0.1343\times exp\left(1.4296\times Vp\right)$$

(3) $$Fc=0.0258\times exp\left(1.9494\times Vp\right)$$

**Figure 8:**
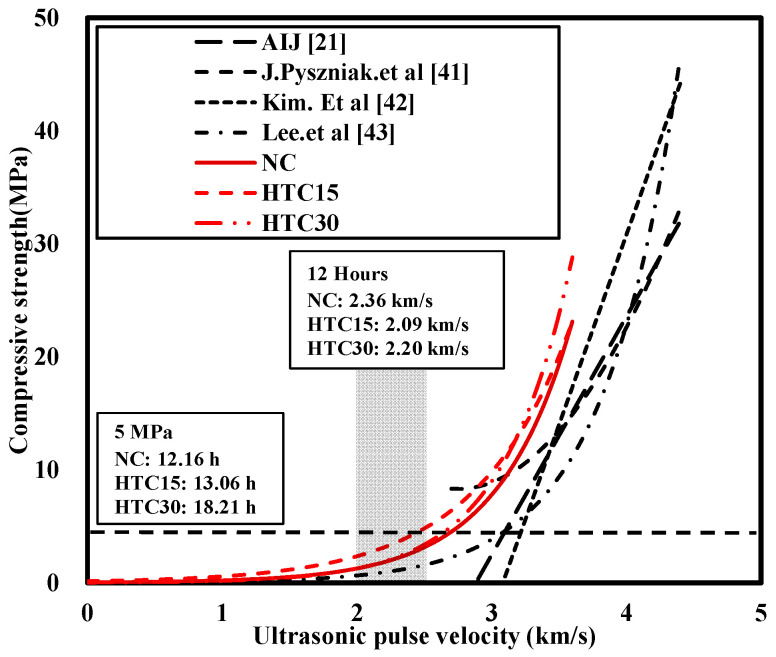
Comparison with existing strength prediction models.

Figure 8 compares the strength prediction model of existing researchers with the strength prediction Equations (1)–(3) proposed in this study. In the case of Formula (1), it is concrete without mixing NHT, in Formula (2) it is concrete mixed with 15% non-NHT, and in Formula (3) it is concrete mixed with 30% NHT. Each equation is expressed through the values of compressive strength and ultrasonic speed at the respective ages of W/B 41% and W/B 33%. At UPV of 3 km/s, NC showed 7.8 MPa, and HTC-15 showed 9.8 MPa, which was 25.6% higher than NC. In addition, HTC-30 showed a 14.1% higher strength at 8.9 MPa, and it can be seen that the compressive strength of concrete mixed with NHT at the same UPV is higher than that of concrete without NHT mixed. Only some of the existing strength prediction models have been tested on initial age, and Figure 8 shows that research on initial age is insufficient. As a result of comparing existing researcher Lee’s strength prediction model with HTC15 and HTC30, HTC15 and HTC30 showed the compressive strength at about twice higher than that of Lee’s strength prediction model at UPV 3 km/s. In this study, the experimental strength prediction equation showed different compressive strengths at the same UPV compared to the remaining prediction models. The reason for the different results is believed to be that the UPV increased as NHT filled the pores between hydration products during the cement hydration process. Additionally, the results are believed to be different because the chemical composition of the cement used in each experiment was different. 

## 4. Conclusions

In this study, the strength of concrete mixed with NHT as a cement substitute was evaluated at early ages using UPV, which is a non-destructive method. In addition, the correlation between compressive strength and UPV was analyzed.

1. The compressive strength of HTC41-15 was 37% higher than that of NC41 after 8 h, but it was 27% lower with a value of 6.58 MPa after 24 h. The compressive strengths of HTC33-15 and HTC33-30 were similar to that of NC33 until 15 and 10 h, respectively. The difference between the strengths of NC33 and HTC33 increased over time.
2. At a water-to-binder (W/B) ratio of 41%, mortar mixed with NHT showed high UPV in contrast to concrete. However, at a W/B ratio of 33%, the UPVs of mortar and concrete were similar. As the NHT substitution proportion and W/B ratio increased, UPV decreased.
3. The correlation between compressive strength and UPV was analyzed. The correlation coefficients (R^2^) for HTC41-30 and HTC33-30 were 0.88 and 0.94, respectively. In contrast, a high correlation coefficient of 0.95 or higher was observed for other specimens. Since the strength prediction formula showed a high correlation coefficient in this experiment, it is judged that it is possible to predict strength at an early age using the strength prediction formula.
4. As a result of comparing the strength prediction formula proposed in this study with the strength prediction models of existing researchers, the trend lines for NC and HTC were similar, but the results were different from the strength prediction models of existing researchers. In addition, it can be seen that the strength prediction models of existing researchers lack research on early age.
5. In this study, Equations (1)–(3) were derived through the correlation between concrete compressive strength and UPV. It is believed that it can be applied to quantitatively evaluate the compressive strength at the initial age of concrete mixed with NHT.

In the scope of this study, a prediction model using compressive strength and ultrasonic speed was presented for the development of initial strength using NHT, but the analysis of hydration mechanism and tissue composition were somewhat lacking. We plan to confirm these aspects through additional research in the future, and through this, we believe that the use of eco-friendly materials such as hwangto will contribute to reducing the damage caused by current global warming.

## Figures and Tables

**Table 1:** Experimental factor.

Contents	Detail
Mortar	Normal mortar (NM)
	Non-sintered hwangto mortar (HTM)
Concrete	Normal concrete (NC)
	Non-sintered hwangto concrete (HTC)
Replacement ratio of non-sintered hwangto	0%, 15%, 30%
Curing	24 h
Mechanical properties	Compressive strength (MPa)
	Ultrasonic pulse velocity (km/s)

**Table 2:** Experimental plan.

Specimen	Mechanical Properties	Measurement Time	
		6–12 h	12–24 h
Concrete	Compressive strength (MPa)	1/120 min	1/120 min
	Ultrasonic pulse velocity (km/s)	1/30 min	1/60 min
Mortar	Ultrasonic pulse velocity (km/s)	1/30 min	1/60 min

**Table 3:** Physical properties of materials.

Materials		Properties
Binder	Cement	Type I ordinary Portland cement, Density: 3.15 g/cm^3^, Fineness 3200 cm^2^/g
	Non-sintered hwangto	Density: 2.50 g/cm^3^, Fineness 3300 cm^2^/g
Coarse aggregate	Crushed granite aggregate	Density: 2.68 g/cm^3^, Fineness modulus: 7.03, Absorption: 0.68%, Maximum size: 20 mm
Fine aggregate	River sand	Density: 2.54 g/cm^3^, Fineness modulus: 2.54, Absorption: 1.6%
Super plasticizer		Polycarboxylic-based acid

**Table 4:** Chemical properties of the normal concrete and non-sintered hwangto concrete.

Materials	Chemical Composition (%)								L.O.I ^(3)^
	CaO	SiO_2_	Al_2_O_3_	Fe_2_O_3_	MgO	SO_3_	K_2_O	Others	
OPC ^(1)^	60.34	19.82	4.85	3.30	3.83	2.88	1.08	0.86	3.02
NHT ^(2)^	0.93	40.0	32.9	7.79	1.54	-	0.76	16.62	13.7

^(1)^ OPC: ordinary Portland cement; ^(2)^ NHT: non-sintered hwangto; ^(3)^ L.O.I: loss on ignition.

**Table 5:** Mix proportions of the NC and HTC.

MIX ID	W/B (%)	S/a (%)	Unit Weight (kg/m^3^)				
			W	C	NHT	S	G
NC41	41.0	46.0	165	400	0	799	956
HTC41-15			165	340	60	794	950
HTC41-30			165	280	120	788	943
NC33	33.0	43.0	165	500	0	711	961
HTC33-15			165	425	75	705	953
HTC33-30			165	350	150	699	944

**Table 6:** SEM images of NM and HTM.

MIX ID	Elapsed Times (Hour)		
	6 h	12 h	24 h
NM	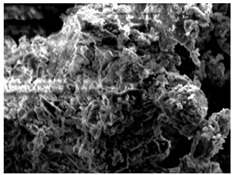	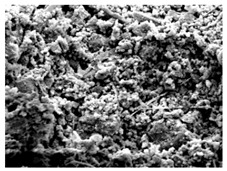	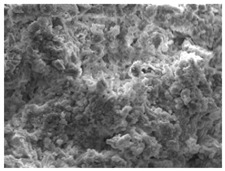
HTM-15	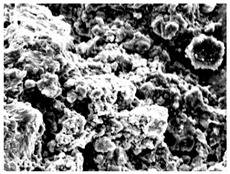	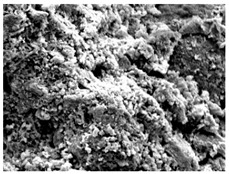	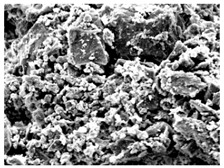
HTM-30	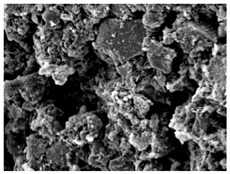	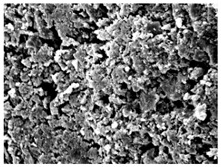	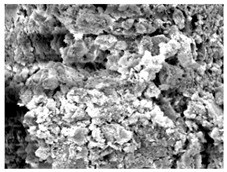

**Table 7:** Prediction model of compressive strength of existing concrete.

Researcher	Predictive Model
AIJ [21]	Fc=21.08×Vp−60.78
J. Pyszniak et al. [41]	$Fc=9.07\times Vp^{2}-49.8\times Vp+76.67$
Kim et al. [42]	Fc=33.53×Vp−103.38
Lee et al. [43]	$Fc=0.0184\times exp\left(1.78\times Vp\right)$

## Data Availability

The data presented in this study are available upon request from the corresponding author.
